# Simulating hybrid energy systems based on complementary renewable resources

**DOI:** 10.1016/j.mex.2019.10.017

**Published:** 2019-10-18

**Authors:** Frederico A. During Filho, Alexandre Beluco

**Affiliations:** Instituto de Pesquisas Hidráulicas (IPH), Universidade Federal do Rio Grande do Sul (UFRGS), Porto Alegre, Rio Grande do Sul, Brazil

**Keywords:** Method to determine the influence of energetic complementarity on the performance of hybrid systems based on complementary resources, Renewable energy, Energetic complementarity, Hybrid energy systems, Computational simulations

## Abstract

The complementarity between energy resources can influence the performance of hybrid generation and storage systems, and can also decisively influence their design. Renewable resources may have intermittent characteristics that make the study of the influence of complementarity on the performance of hybrid systems quite difficult. The establishment of a performance limit of hybrid systems based on renewable resources and the study of the effects of complementarity considering this limit can provide interesting results. This performance limit can be established with an idealization of the mathematical functions describing the energy availability of the explored renewable resources. This article presents a method for analyzing the performance of hybrid systems based on complementary resources. The method allows to evaluate the influence of different levels of complementarity between the exploited resources on the cost of energy and capacity shortage. Utilizing idealized energy availability, the result sets a performance limit.

• A method to evaluate the impact of complementarity on the performance and reliability of hybrid systems.
• The energy availabilities of the renewable sources are idealized and allow the characterization of a limit of performance.
• Different levels of complementarity can be related with design parameters of hybrid energy and storage systems.

## Graphical abstract

**Figure:**
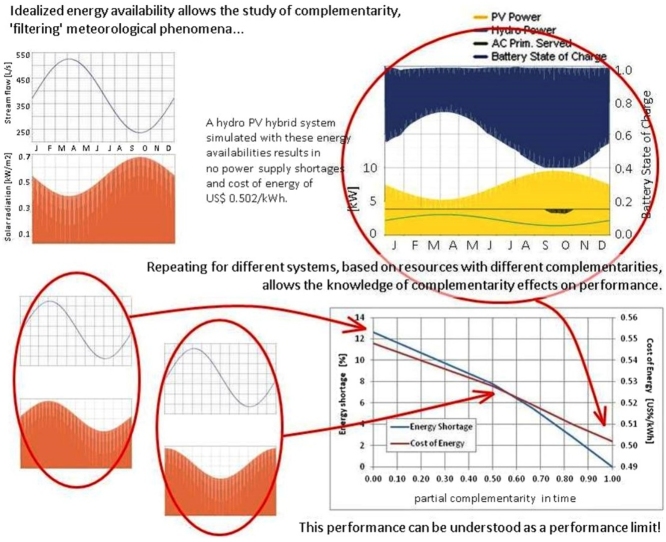

**Specification Table**

**Table:** 

Subject area:	*Energy*
More specific subject area:	*Renewable Energy – Hybrid Energy Systems*
Method name:	*Method to determine the influence of energetic complementarity on the performance of hybrid systems based on complementary resources*
Name and reference of original method:	*A method to evaluate the effect of complementarity in time between hydro and solar energy on the performance of hybrid hydro PV generating plants. Beluco et al.* [1]*. Renewable Energy (2012), v. 45, p. 24–30.*
Resource availability:	*N/A*

## Background

Predicting renewable energy availability is difficult because of its typical variability, and planning its technical and economic feasibility becomes a daunting task. Possible complementarity between energy resources may contribute to better project viability, but the characteristic variability of renewable resources makes it difficult to assess the effects of complementarity on the performance and reliability of hybrid systems based on complementary resources. Knowing better the effects of complementarity on the performance of hybrid systems then becomes necessary.

The work of Beluco et al. [2] discussed the concept of complementarity and proposed a method [3] to evaluate temporal complementarity. Years later, the work of Risso et al [4] explored the concept of complementarity between renewable energy resources located in different locations and proposed a method [5] to evaluate spatial complementarity. The review article by Jurasz et al. [6] presents a fairly complete survey of the articles related to complementarity published in the last years and brings a definitive discussion about the concept of energetic complementarity.

The paper by Beluco et al. [1] proposes a method allowing access to the effect of complementarity on a hybrid system using an idealized energy availability function instead of natural raw data. The idea is to extract variability and intermittent effects from renewable resources and work with smooth mathematical functions. This method deals with an idea of performance limit [7] that could be associated with an idealization of raw data that would be difficult to reach in real conditions. This concept of 'performance limit' can be improved to the concept of 'idealized performance', which would be a hybrid system performance in response to a given series of idealized data. For example, a hydro PV hybrid system can be studied from data idealized by average energy availabilities, but also by maximum values and minimum values, leading to different technical and economic performance results. A still open question is the relationship between how to idealize raw data and the effects of complementarity to be explored. This article presents the method proposed by Beluco et al. [1] in a more generic and more appropriate way for the direct application in research activities and in engineering projects.

The next section describes the method to determine the influence of energetic complementarity on the performance of hybrid systems based on complementary energy resources, indicating at each step comments that clarify the actions to be performed based on results discussed in Refs. [1] and [8]. Ref. [9] also presents similar results.

## Method details

**Fig. 1:**
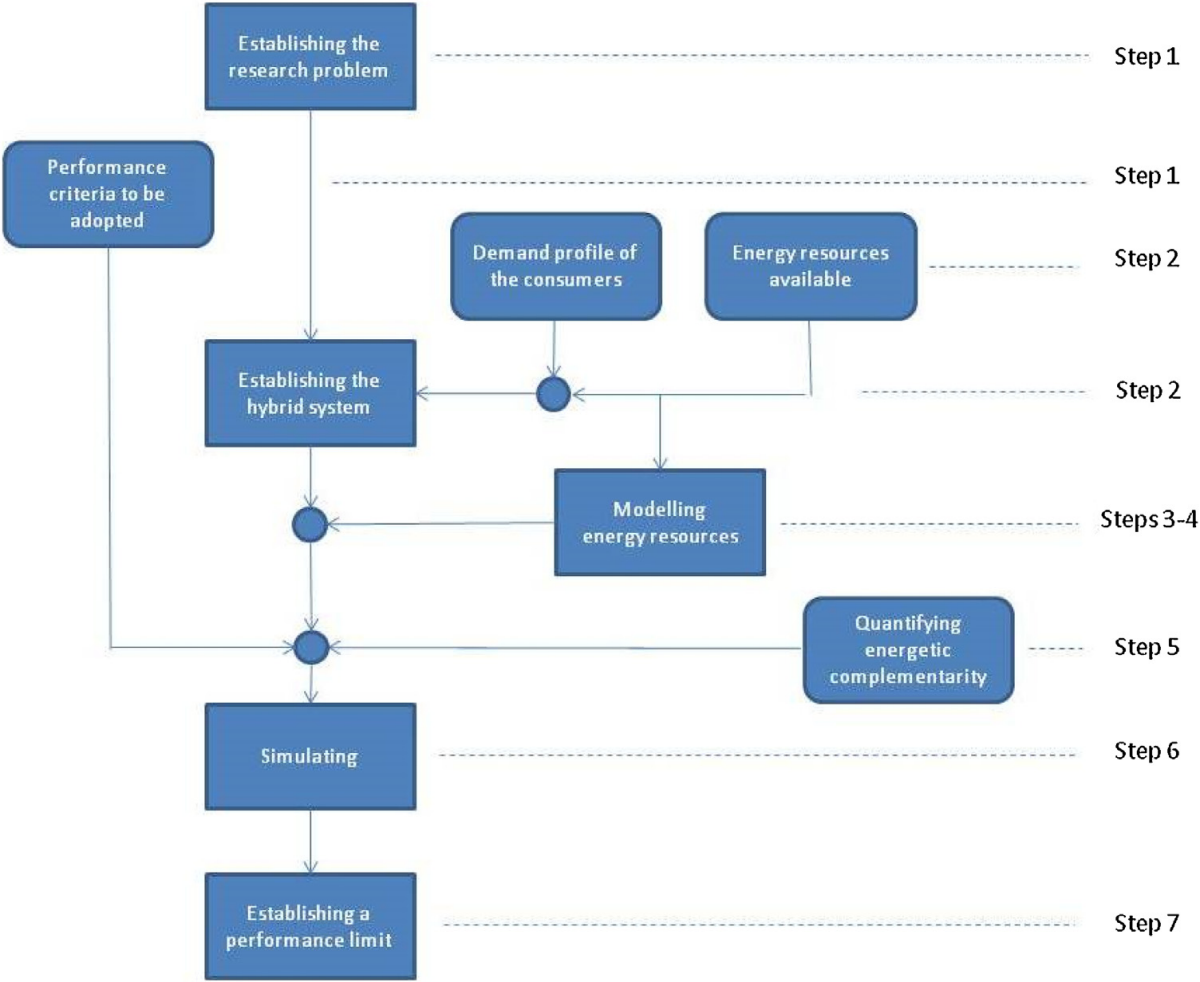
Flowchart for the proposed method to simulate hybrid systems based on complementary renewable resources, establishing a performance limit.

The method for simulating hybrid energy systems based on complementary renewable resources consists of the following seven steps, accompanied with notes detailing each step with comments extracted from Refs. [1] and [8]. The flowchart in Fig. 1 shows a scheme with these steps and clearly indicates the data to be obtained and the decisions to be made throughout the process. The next section exemplifies the application of this method based on the results of Refs. [1] and [8].

The objective of this method is to allow the study of the effect of complementarity on the performance of hybrid systems. The effect of complementarity is studied with the idealization of energy availability, filtering the typical variability of renewable resources. Thus, i a performance limit of systems based on renewable resources with some degree of complementarity is estimated.

**Fig. 2:**
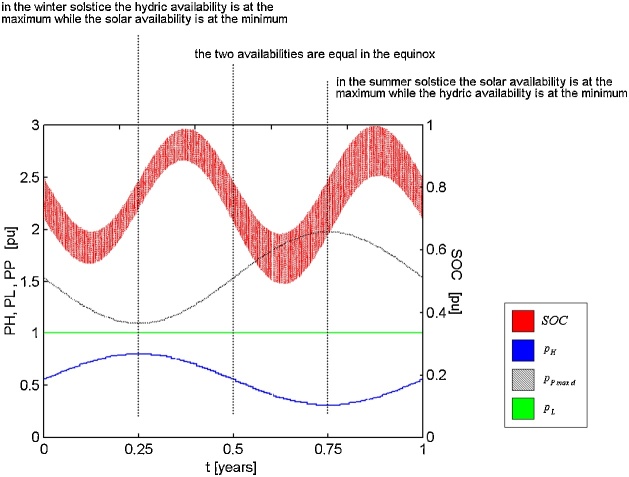
Results of the simulation of a hydro PV hybrid system as shown in Ref. [1]. Conventions: SOC: state of charge of batteries, p_H_: power generated by the hydro generator, p_p_: maximum daily power from the PV generator, p_L_: power supplied to the loads.

**Fig. 3:**
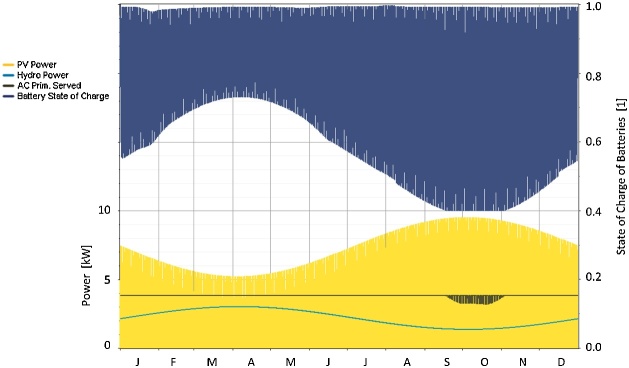
Results of the simulation of a hydro PV hybrid system as shown in Ref. [1].

**Fig. 4:**
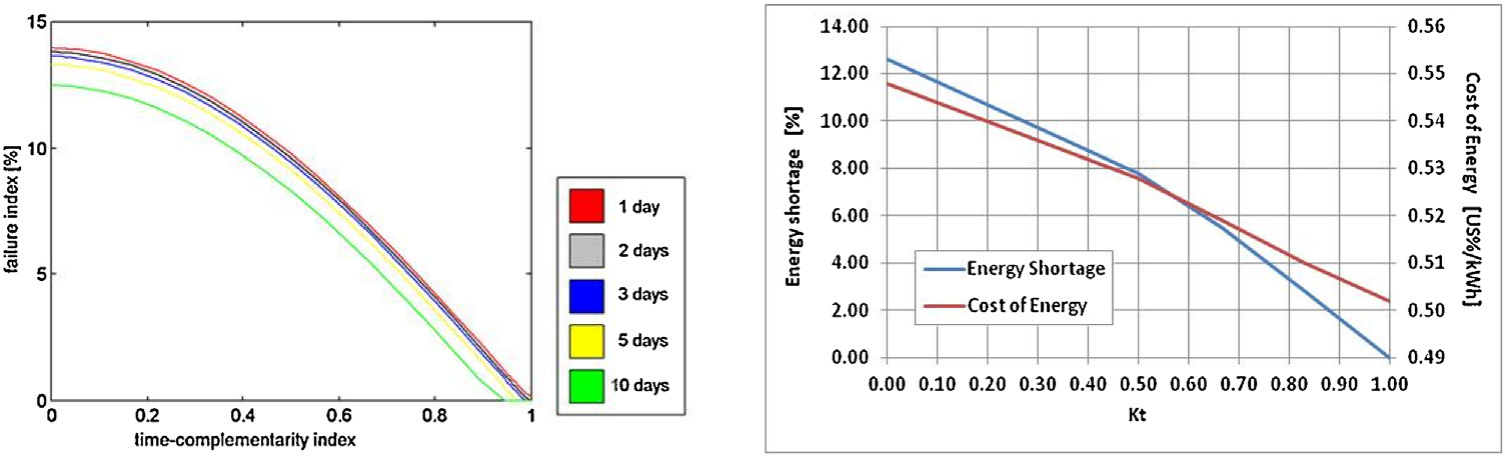
Failure index as a function of time-complementarity index, as shown in Ref. [1], on the left, with the caption indicating the number of days of battery capacity, and energy shortage (in blue) and cost of energy (in red) as a function of the time-complementarity index (κt), as shown in Ref. [8], on the right.

The method consists of the following steps:

1 Establish the research problem related to the influence of complementarity on the performance of hybrid systems and delimit the relations between complementarity and performance to be determined
  Note - Ref. [1] seeks to determine the relationship between energetic complementarity and system failure in energy supply to consumers, while Ref. [8] aims to determine the influence of complementarity on excess energy and on the cost of energy.
2 Establish the components of the hybrid system to be studied and the renewable energy resources to be used by this system, characterizing the relationship between the energy supplies demanded by consumers and the available energy sources.
  Note - The hybrid energy systems discussed in Refs. [1] and [8] are hydro PV hybrid systems. Both systems contain, in addition to hydroelectric power plant and photovoltaic modules, converters for energy flow between DC and AC bus bars and batteries for energy storage, serving consumers in AC. Component sizing will be the result of an optimization process to reduce total net present cost over the 25-year analysis period.
3 Determine parameters of the renewable resources used to obtain energy supplies that allow their modeling, such as average, minimum and maximum values and their variability or intermittent characteristics.
  Note - Ref. [1] uses p.u. system, but Ref. [8] considers a system with a hydroelectric plant with 2.22 kW installed, with 17.532 kW in photovoltaic modules, with batteries of various dimensions. The water availability has an average value of 0.380 m^3^/s, with a minimum of 0.240 m^3^/s and a maximum of 0.521 m^3^/s. Solar availability presents an average value of 3.815 kW h/m^2^/day, with a minimum of 1.940 kW h/m^2^/day and a maximum of 5.922 kWh/m^2^/day.
4 Define the appropriate models for describing the hourly values (according to the time base adopted in the analysis) of the energy availability of the renewable resources used, usually basic mathematical or probabilistic functions.
  Note 1 - In both cases, Refs. [1] and [8], the simulations will be carried out for a period of one year, with steps of one hour. The energy availabilities will be idealized with the use of sinusoidal functions. Water availability was simulated with only one sinusoidal function. Solar availability was simulated using three sine-wave functions and can be simulated with specific models, such as those presented for example by Duffie and Bekman [10].
  Note 2 - The composition of mathematical models to describe each renewable energy resource is still an open topic. Describing the availability of hydropower and solar power with sine curves is relatively easy. However, choosing a model that describes the availability of wind energy or wave energy with a similarly generic characteristic is not so easy.
5 Establish the most appropriate method for the quantification of energetic complementarity, considering the relationship between available power and energy required and the complementarity aspects to be studied.
  Note - Energetic complementarity was quantified, both in Refs. [1] and [8], with the dimensionless index proposed by Beluco et al. [2,3] In Refs. [1] and [8], only the time component of temporal complementarity was considered.
6 Execute the simulations with a suitable platform (for example, MatLab [11], Homer [12] etc.) and apply appropriate criteria to evaluate the effects of complementarity on the performance of the hybrid systems studied
  Note - In Ref. [1] the simulations were performed with an older version of MatLab (with one result shown in Fig. 2) and the criterion adopted was the failure rate in the energy supply to consumers. In Ref. [8] the simulations were done with the software Homer, Legacy version, (with a result is shown in Fig. 3) and the adopted criteria were the energy shortage and cost of energy. A good criterion would be reliability [13].

(Reproduced from Ref. [1]).

(Recreated from Ref. [8]).
7 Establish the results, setting a performance limit to the hybrid system that has been studied, an unattainable limit since the available energy considered were idealized.
  Note - Ref. [1] presented the final result in a graph showing failure index as a function of time-complementarity index, while Ref. [8] presented energy shortage and cost of energy as a function of the time-complementarity index. Fig. 4 shows these results, establishing the limits of performance in each case.

Fig. 1 shows the steps of the method which make up its backbone, with rectangles with sharp corners. In the first step, the research problem should be established by defining the type of hybrid system to be studied, the complementarity component that will be considered in this study and the parameter that will be used to evaluate the performance of the hybrid system.

In the second step, the hybrid system is established, knowing the demand profile of consumers and the energy resources available to supply the demand. The hybrid system may also contain or shall contain rectifiers, inverters, fossil fuel based support systems and energy storage devices. In the third and fourth steps, the idealization of energy resources must be established.

The sixth step is the simulation of the hybrid system and the seventh step includes the evaluation of the results. The scaling of hybrid system components is based on energy availability and of course these components will be 'larger' than equivalent systems suitable for non-ideal situations. These simulations will be repeated as many times as necessary to compose the intended result in the final step. The conclusion of the simulations for different complementarities will allow gathering the data obtained in a final and conclusive relationship between the parameter adopted to evaluate the hybrid system performance and the index chosen to quantify the energetic complementarity.

Fig. 1 also shows the data to be obtained or elaborated for the application of the method in rectangles with rounded corners. In the first step, the parameter that will be used to evaluate system performance needs to be determined. This parameter can be the energy cost or the amount or total time of power supply failures to consumers.

In the second step, the resources available for energy conversion and the consumer demand profile should be established. Renewable energy resources should be modeled to reproduce their characteristic natural cycles and to 'filter out' the effects of climatological phenomena. In the fifth step, the method for quantifying energetic complementarity should be established. The articles by Beluco et al. [2,3] suggest a dimensionless index, but there are other methods available in the literature.

### Examples of application

The results presented in Refs. [1] and [8] can be considered as examples of application of the method proposed in this article. These two references, with some slight differences between them, present studies in which photovoltaic hydroelectric hybrid systems were simulated with water and solar resources presenting different degrees of complementarities. Both articles aimed to study the influence of complementarity on the performance of hybrid systems.

The study presented in Ref. [1] composes the article that originally presented the idea of this method. This study presents simulations (performed with MatLab software [11]) of a photovoltaic hybrid hydroelectric system, with energy storage in batteries, supplying direct current consuming loads. Water and solar availabilities were idealized and different complementarities were created artificially, varying characteristics of these availabilities. The simulations were performed determining in each case the events of power supply failure to consumers.

Fig. 2 shows one of the results from Ref. [1] corresponding to a system with full complementarity. The blue line shows the power provided by the hydropower plant and the black line shows the maximum power values provided by the photovoltaic modules over the days of the year. The green line shows the demand of consumer cargo, considered constant throughout the days. The red line shows the instantaneous state of charge (SOC) of the batteries over the days of the year and this line looks like a blur because of the daily variation of the state of charge over the days.

Note that, in this case, the energetic complementarity is full and the minimum availability of the two energy resources occur with six months of lag between them. Thus, the minimum availability value of one resource coincides with the maximum value of the other. The battery bank has been scaled to hold up to two days of total power supply to consumer loads, and with this storage capacity the operation of the hybrid system has resulted in flawless supply over a year. Also note the behavior of the state of charge of the batteries over the year, with two valleys and two peak loads between minimum charge and maximum charge.

The work of Ref. [8] applies this method to a hybrid system in much the same way as Ref. [1], now using Homer software. A hybrid hydropower photovoltaic system is simulated for all combinations of optimization variables as described by Refs. [14] and [15]. Then the simulations are repeated for the sensitivity variables. Unlike the study presented in Ref. [1], in this case the simulations take into consideration the costs of the system components. This system also contains energy storage in batteries. The simulations were also repeated for energy availability with different degrees of complementarity and in each of them cost of energy and annual capacity shortages were determined.

Fig. 3 shows one of the results from Ref. [8] similar to the result shown in Fig. 2, corresponding to a system with full complementarity. The light blue line shows the energy supplied by the hydroelectric power plant and the yellow spot shows the energy supplied by the photovoltaic modules. The black line shows the demand of consumer loads, also considered constant throughout the days. The dark blue line shows the instantaneous state of charge of the batteries over the days of the year and this line also looks like a blur due to the daily change in the state of charge.

Finally, Fig. 4 shows a summary of the final results of Ref. [1] on the left and Ref. [8] on the right. The graph on the left shows the variation of the failure rate as a function of the complementarity index over time, naturally indicating that power supply failures decrease with better complementarities. The graph on the right shows cost of energy and capacity shortage as a function of the time complementarity index. In these graphs, these two variables show reductions due to better complementarities, and even these two curves intersect as the cost of energy tends to decrease for systems that accept major power supply failures.
